# Epidemiological and clinical characteristics of three family clusters of COVID-19 transmitted by latent patients in China

**DOI:** 10.1017/S0950268820001491

**Published:** 2020-07-06

**Authors:** Jing Li, Jiguang Ding, Li Chen, Liang Hong, Xiaoqi Yu, Enling Ye, Gangqiang Sun, Binbin Zhang, Xinxin Zhang, Qingfeng Sun

**Affiliations:** 1 Research Laboratory of Clinical Virology, Ruijin Hospital, Shanghai Jiaotong University School of Medicine, Shanghai, China; 2 Department of Infectious Diseases, Ruian People's Hospital, Ruian, Zhejiang, China; 3 Department of Gastroenterology and Hepatology, Ruijin Hospital North, Shanghai Jiaotong University School of Medicine, Shanghai, China; 4 Clinical Research Center, Ruijin Hospital North, Shanghai Jiaotong University School of Medicine, Shanghai, China; 5 Department of Endocrinology, Ruian People's Hospital, Ruian, Zhejiang, China; 6 Department of Biology, Gordon College, MA, USA; 7 Medical Administration Department, Ruian People's Hospital, Ruian, Zhejiang, China

**Keywords:** COVID-19, epidemiology, family cluster, symptom assessment

## Abstract

From 21 January 2020 to 9 February 2020, three family clusters involving 31 patients with coronavirus disease 2019 were identified in Wenzhou, China. The epidemiological and clinical characteristics of the family cluster patients were analysed and compared with those of 43 contemporaneous sporadic cases. The three index cases transmitted the infection to 28 family members 2–10 days before illness onset. Overall, 28 of the 41 sporadic cases and three of 31 patients in the family clusters came back from Wuhan (65.12 *vs.* 9.68%, *P*< 0.001). In terms of epidemiological characters and clinical symptoms, no significant differences were observed between the family cluster and sporadic cases. However, the lymphocyte counts of sporadic cases were significantly lower than those of family cluster cases ((1.32 ± 0.55) × 10^9^/l *vs.* (1.63 ± 0.70) × 10^9^/l, *P* = 0.037), and the proportion of hypoalbuminaemia was higher in sporadic cases (18/43, 41.86%) than in the family clusters (6/31, 19.35%) (*P* < 0.05). Within the family cluster, the second- and third-generation cases had milder clinical manifestations, without severe conditions, compared with the index and first-generation cases, indicating that the virulence gradually decreased following passage through generations within the family clusters. Close surveillance, timely recognition and isolation of the suspected or latent patient is crucial in preventing family cluster infection.

## Introduction

Since December 2019, an epidemic of coronavirus disease 2019 (COVID-19), associated with severe acute respiratory syndrome coronavirus 2 (SARS-CoV-2), emerged in Wuhan, Hubei province, China [1, 2]. SARS-CoV-2 has been characterised by high infectivity through human-to-human transmission and relatively low mortality [3]. The mean R0 of SARS-CoV-2 is estimated to range from 2.24 to 3.58 [4, 5]. As a result, the epidemic of COVID-19 has rapidly spread to the whole country and worldwide. As of 26 March 2020, 195 countries had been affected, and the cumulative number of confirmed cases had reached 81 968 and 389 199 in China and worldwide, with 3293 and 17 914 deaths, respectively.

Currently, the epidemiological characteristics of COVID-19, especially transmission patterns, have not been well elucidated. The first family clustering study reported that five family members who travelled to Wuhan from Shenzhen were infected with SARS-CoV-2, and when they returned to Shenzhen, the additional family members who did not travel to Wuhan became infected with the virus [6]. The epidemiological and phylogenetic analysis indicates that SARS-CoV-2 can be transmitted person-to-person in hospital and family settings [6]. Subsequently, several studies also reported the family cluster transmission resulting in the infection of 3–11 family members [7–11], even during the incubation period. However, further investigation is required to understand the transmission patterns among family members.

Wenzhou is one of the regions with a high prevalence of infection outside Hubei province, probably because of the close economic cooperation and convenient public transportation between two regions. In the early stage of the epidemic, over 7000 people came back to Ruian, a county-level city under the administration of Wenzhou. Among these people, 74 were diagnosed with COVID-19, and three family clusters of 31 members were identified.

In this study, we aimed to investigate the epidemiological and clinical characteristics of these three family clusters of COVID-19 cases by comparing them with sporadic cases, which would provide insights for epidemic control in the context of the current serious situation worldwide.

## Patients and methods

### Patients

**Fig. 1.**
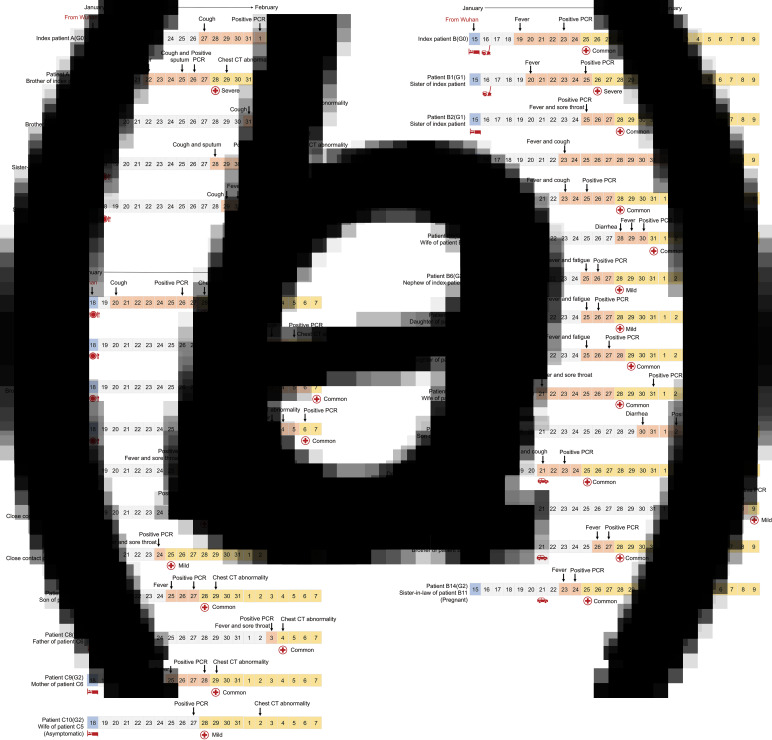
Timeline of close contacts, illness onset, polymerase chain reaction (PCR) test or computed tomography (CT) and hospitalisation for family clusters a, b and c during 15 January 2020 and 9 February 2020. Patient numbering is based on kindred relationship. (a) Index case A (G0) had dinner with four cases A1–4 (G1). It is noticed that the time of illness onset of case A1 (G1) was earlier than that of index case A (G0); (b) Index case B (G0) had close contact with four cases B1, 2, 10 and 11 (G1). B2 (G1) transmitted the infection to six cases B3–6 and 9 (G2) and B11 (G1) transmitted the infection to cases B12–14 (G2). B6 (G2) transmitted the infection to two cases B7–8 (G3); (c) Index case C had a party with six cases C1–6 (G1). Case C6 (G1) transmitted the infection to three cases C7–9 (G2), and case C5 (G1) transmitted the infection to case C10 (G2).

**Fig. 2.**
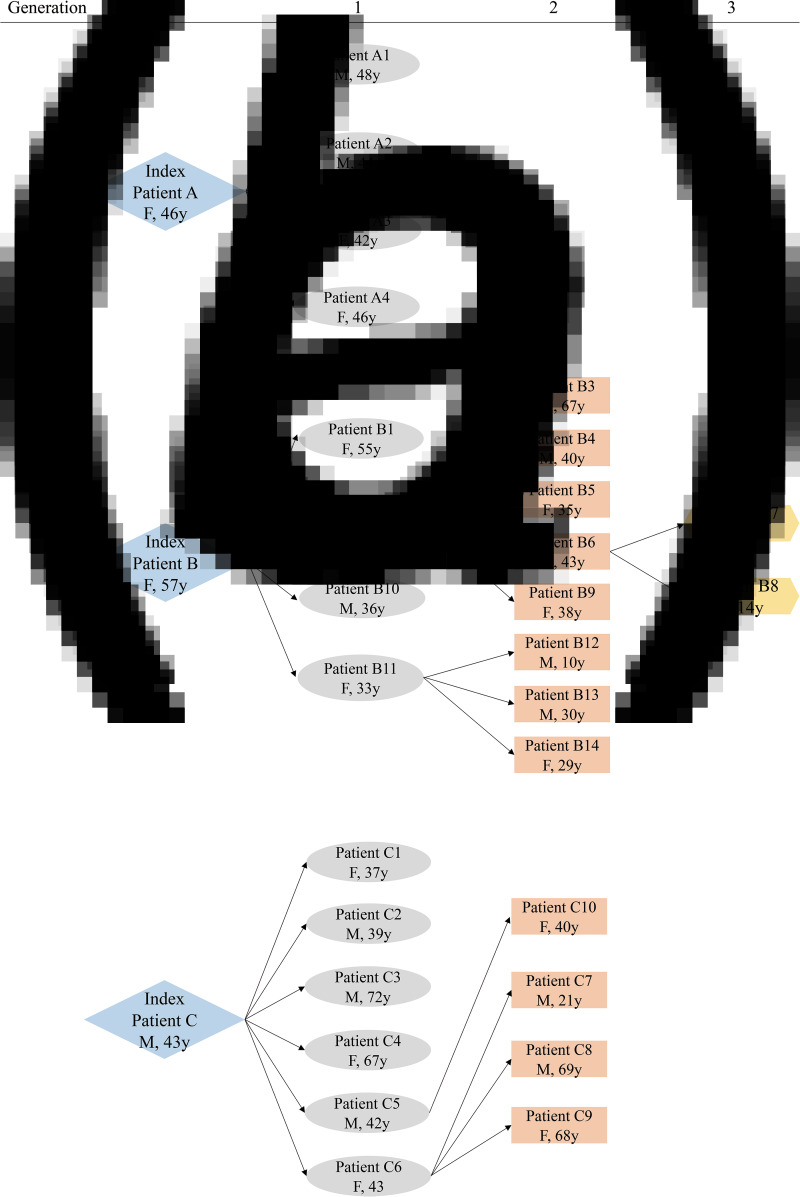
The generation to the index cases in family clusters a, b and c.

From 21 January 2020 to 9 February 2020, 74 COVID-19 patients, who were all positive for the nucleic-acid test of SARS-CoV-2 and received isolation and treatment in the designated Ruian People's Hospital, were enrolled in the present retrospective study. Among the patients, 31 patients were identified to belong to three different families according to their family relationships and history of close contacts (Figs 1 and 2), while the other 43 patients were sporadic cases.

Symbols (meal , vehicle , live together , cleaning ): date of close contact; symbol (facing down arrow ): date of symptom onset, or PCR test/CT scan; symbol (red cross ): date of hospitalisation. Behind red cross symbol is clinical type at the admission.

The epidemiological history, including exposure history, travelling vehicle, contact tracing, family member relationship, date of illness onset and date of admission and isolation, were collected in detail for each patient by two attending physicians. The transmission chain was carefully evaluated to clarify the relationship within the cluster members, according to the close contact history and exposure time.

A close contact was defined as an act of sharing a meal, party, vehicle or living room with a confirmed or latently infected patient within 14 days. An index case (G0) was defined as the original source of SARS-CoV-2 infection among the family. The patients who were infected by contact and exposure to the index case were defined as the first-generation cases (G1), and the patients who were infected by contact and exposure to the first-generation patients were defined as second-generation cases (G2) and so on. The patients without an infected family member were defined as sporadic cases.

Index case A, a 46-year-old female who came back from Wuhan on 17 January 2020, had dinner with four family members on 18 January 2020. She developed symptoms of cough 10 days later and was confirmed with COVID-19 on 1 February 2020. The four family members, cases A1–4 (G1), developed the disease 5−14 days after the dinner and were called first-generation cases of index case A (Figs 1a and 2a).

Index case B was a 57-year-old female who came back from Wuhan on 15 January 2020. She developed a fever 4 days later and was diagnosed with COVID-19 on 23 January 2020. During the next two weeks, four family members, cases B1, 2, 10 and 11 (G1), who had a close contact history with index case B were confirmed to be infected by SARS-CoV-2 sequentially. Then, case B2 (G1), one of the four first-generation cases of index case B, transmitted the infection to five family members, cases B3–6 and 9 (G2), who were called second-generation cases. Another first-generation case, B11 (G1), transmitted the infection to three family members, B12–14 (G2). Moreover, one of the second-generation cases, B6 (G2) transmitted the infection to cases B7–8 (G3), the third-generation cases (Figs 1b and 2b).

Index case C (G0), a 43-year-old male, came back from Wuhan and joined a party with four family members and two classmates on 18 January 2020. He developed illness with cough 2 days later and was diagnosed with COVID-19 on 26 January 2020. The six persons, cases C1–6 (G1), who joined the party also developed an illness within the next 2 weeks. Case C6 (G1) was responsible for the second-generation infection to three family members, cases C7–9 (G2), while case C5 (G1) was responsible for transmission to one family member, C10 (G2) (Figs 1c and 2c).

All patients were diagnosed with COVID-19 by real-time reverse transcriptase-polymerase chain reaction (RT-PCR) assay, according to the Guideline for Diagnosis and Treatment for Novel Coronavirus Pneumonia released by the National Health Commission of China (5^th^ edition) [12]. The RT-PCR tests for influenza A and B for all patients were negative. Written informed consent, according to the Declaration of Helsinki, was obtained from each patient. This study was approved by the Ethics Committee of the Ruian People's Hospital (Approval number: YJ20200013).

### Data collection

The clinical information of all enrolled patients was retrieved from the hospital history system, including the demographic data, laboratory test results, radiological results, treatment regimens, duration of treatment, duration of hospitalisation and treatment outcomes. The applications of intranasal oxygen inhalation and assisted mechanical ventilation along with comorbidities including hypertension, diabetes, chronic obstructive pulmonary disease, chronic kidney disease and malignant tumours were recorded.

The patients were divided into different clinical types, according to the Guidelines by the National Health Commission of China [12]. Patients who presented with classic symptoms and positive SARS-CoV-2 RNA but without pneumonia lesions on computed tomography (CT) scan were defined as mild cases, and those with classic symptoms, positive SARS-CoV-2 RNA and pneumonia lesions on CT scan were defined as common cases. In addition, patients who met the following criteria were defined as severe cases: (1) respiratory distress, a respiratory rate (RR) ≥30 beats/min; (2) an oxygen saturation level less than 93% in resting state and (3) a partial pressure of oxygen (PaO_2_)/oxygen concentration (FiO_2_) ≤300 mmHg (1 mmHg = 0.133 kPa).

### SARS-CoV-2 RNA detection

SARS-CoV-2 RNA was detected by RT-PCR assay with the Taqman probes targeting ORF1ab, N and E genes, and expressed as the cycle threshold (Ct) value (Shanghai BioGerm Medical Biotechnology Co., Ltd). The amplification products for genes with a Ct value of less than 38 were considered as positive. Sputum samples or throat swab samples were taken for analysis at baseline and then every 2–3 days until hospital discharge.

### Statistical analysis

Continuous variables were expressed as mean and standard deviation (SD) or median and interquartile range (IQR), and categorical variables were expressed as a number (%). The values were compared by Student's *t*-tests, one-way ANOVA or Mann−Whitney test, or Kruskal−Wallis as appropriate. All data analysis was performed with R software (version 3.6.2) and EmpowerStates software (http://www.empowerstats.com, X&Y solutions, Inc., Boston, MA). A two-sided *P* value of less than 0.05 was considered statistically significant.

## Results

### General demographic and clinical characteristics

Of all 74 patients, 35 were male, and 39 were female. The mean age was 44.26 years old. The most common symptoms were fever (83.78%), cough (78.38%) and sputum (52.70%). The most frequent comorbidity was hypertension (14.86%). Most patients (90.54%) were non-smokers. Seventy (94.59%) patients were mild (*n* = 15) or moderate (*n* = 55) cases, while only four (5.4%) patients had severe pneumonia (A1 (G1), B1 (G1) and two sporadic cases). At admission, varying degrees of pulmonary lesions were presented in 59 (79.73%) patients as detected by CT scan (Table 1).

**Table 1.** Demographic and clinical characteristics of sporadic and family cluster cases with COVID-19

	Overall (*n* = 74)	Sporadic cases (*n* = 43)	Family cases (*n* = 31)	*P* value
Age (years)	45.26 ± 15.68	47.16 ± 14.69	42.61 ± 16.85	0.22
Sex (M/F)	35/39	21/22	14/17	0.755
From Wuhan (yes/no)	31/43	28/15	3/28	<0.001
Incubation period (days)	5.00 (4.00–7.00)	4.00 (2.00–7.00)	6.00 (4.00–7.00)	0.192
Time from symptom onset to hospitalisation (days)	3.00 (2.00–5.00)	3.00 (2.00–5.00)	3.00 (2.50–5.50)	0.948
Time from symptom onset to diagnosis (days)	3.00 (2.00–5.00)	3.00 (2.00–4.50)	3.00 (2.00–5.00)	0.51
Duration of hospitalisation (days)	14.00 (10.00–17.00)	14.00 (10.00–17.00)	14.00 (10.50–17.50)	0.837
Viral load (Ct value)	30.05 ± 4.58	30.55 ± 4.78	29.46 ± 4.37	0.38
ALT (U/l; range: 9–50)	19.00 (14.25–34.50)	18.00 (13.50–38.00)	22.00 (15.00–31.00)	0.893
AST (U/l; range: 15–40)	23.00 (18.25–33.00)	23.00 (18.50–32.50)	23.00 (18.50–33.00)	0.749
Albumin (normal/decreased)	50/24	25/18	25/6	0.041
Creatinine (μmol/l; range 57–110)	64.84 ± 10.83	64.51 ± 10.69	65.29 ± 11.18	0.763
LDH (U/l; range: 120–250)	224.49 ± 66.37	224.84 ± 63.53	224.00 ± 71.19	0.958
CK (U/l; range: 50–310)	79.50 (50.75–105.00)	76.00 (51.50–104.00)	90.00 (51.00–105.00)	0.478
WBC (× 10^9^/l; range: 3.5–9.5)	4.75 ± 1.73	4.55 ± 1.91	5.04 ± 1.44	0.235
Lym (× 10^9^/l; range: 1.1–3.2)	1.45 ± 0.63	1.32 ± 0.55	1.63 ± 0.70	0.037
Neu (× 10^9^/l; range: 1.8–6.3)	2.55 (1.80–3.48)	2.30 (1.60–3.60)	2.90 (2.25–3.35)	0.647
PLT (× 10^9^/l; range: 125–350)	185.50 (149.25–221.75)	184.00 (147.50–220.00)	190.00 (156.50–239.50)	0.51
CD4^+^T cell (/μl; range: 410–884)	569.00 (430.75–761.25)	588.00 (428.50–757.00)	547.00 (433.00–768.00)	0.861
CD8^+^T cell (/μl; range: 190–658)	354.00 (256.25–442.75)	337.00 (240.00–415.50)	405.00 (326.00–460.00)	0.649
NK (/μl; range: 90–536)	319.50 (199.00–412.25)	340.00 (193.00–428.00)	285.00 (205.00–391.00)	0.804
Hypertension	11 (14.86%)	7 (16.28%)	4 (12.90%)	0.687
Diabetes	7 (9.46%)	6 (13.95%)	1 (3.23%)	0.12
Digestive system disease	4 (5.41%)	2 (4.65%)	2 (6.45%)	0.735
Respiratory system disease	2 (2.70%)	2 (4.65%)	0 (0.00%)	0.223
Cardiovascular diseases	1 (1.35%)	1 (2.33%)	0 (0.00%)	0.393
Malignancy	1 (1.35%)	0 (0.00%)	1 (3.23%)	0.236
Smoking	7 (9.46%)	3 (6.98%)	4 (12.9%)	0.671
Symptoms at admission				
Fever	62 (83.78%)	37 (86.05%)	25 (80.65%)	0.534
Cough	58 (78.38%)	34 (79.07%)	24 (77.42%)	0.865
Sputum	39 (52.70%)	22 (51.16%)	17 (54.84%)	0.755
Chest tightness	12 (16.22%)	6 (13.95%)	6 (19.35%)	0.534
Top body temperature (^o^C)	38.12 ± 0.76	38.19 ± 0.73	38.02 ± 0.79	0.362
Severe pneumonia	4 (5.41%)	2 (4.65%)	2 (6.45%)	0.735
Pneumonia at admission	59 (79.73%)	35 (81.40%)	24 (77.42%)	0.675
Clinical type (mild/common/severe)	15/55/4	8/33/2	7/22/2	0.848
ARDS	4 (5.41%)	2 (4.65%)	2 (6.45%)	0.735
Oxygen therapy	44 (59.46%)	25 (58.14%)	19 (61.29%)	0.785
Mechanical ventilation	2 (2.70%)	1 (2.33%)	1 (3.23%)	0.814

ALT, alanine aminotransferase; AST, aspartate aminotransferase; LDH, lactate dehydrogenase; CK, creatine kinase; WBC, white blood cell; Lym, lymphocytes; Neu, neutrophils; PLT, platelets; NK, natural killer cell; ARDS, acute respiratory distress syndrome.

### Comparison of the epidemiological and clinical characteristics between the family cluster and sporadic cases

Of the 43 sporadic cases, 28 (65.12%) returned from Wuhan while three (9.68%) of the family cluster cases returned from Wuhan (*P* < 0.001). The family members were infected by the three index cases who were in the latent period 2–10 days before the onset of illness. Interestingly, the time of illness onset of patient A1, who had no travel history to Wuhan or contact with other patients, was earlier than that of index case A. Patient A1 (G1) developed severe pneumonia subsequently. The incubation period of sporadic cases (4.00 (2.00–7.00) days) was similar to that of the family cluster (6.00 (4.00–7.00) days) (*P* = 0.192). The time from symptom onset to hospitalisation, the time from symptom onset to diagnosis and the duration of hospitalisation were not significantly different between sporadic and family cluster cases (all *P* > 0.05) (Table 1).

There was no significant difference in the frequency of common symptoms, including fever (86.05 *vs.* 80.65%), cough (79.07 *vs.* 77.42%) and sputum (51.16 *vs.* 54.84%), between sporadic and family cluster cases (all *P* > 0.05). Also, the proportions of mild, common and severe types were similar between sporadic (18.60, 76.74, and 4.65%, respectively) and family cluster (22.58, 70.97 and 6.45%) cases (*P* = 0.848). However, the decrease of albumin was more frequent in sporadic cases (41.86%) than in the family cluster cases (19.35%) (*P* < 0.05). While the levels of alanine transaminase, aspartate aminotransferase and creatinine were not different between the two groups (all *P* > 0.05), the level of lymphocyte counts was significantly lower in sporadic cases ((1.32 ± 0.55) × 10^9^/l) than in the family cluster cases ((1.63 ± 0.70) × 10^9^/l) (*P* = 0.037). The viral load (Ct value) was not different between the two groups ((30.55 ± 4.78) *vs.* (29.46 ± 4.37), *P* = 0.38) (Table 2). The imaging features of the pulmonary lesions on CT scan were not apparently different between the two groups (data not shown).

**Table 2.** Demographic and clinical characteristics between generations within the family clusters

	G0 + G1 (*n* = 17)	G2 + G3 (*n* = 14)	*P* value
Age (years)	44.00 (42.00–55.00)	37.00 (23.00–42.25)	0.039
Sex (M/F)	7/10	7/7	0.623
Incubation period (days)	4.50 (4.00–7.75)	7.00 (5.00–7.00)	0.619
Time from symptom onset to hospital (days)	4.00 (3.00–6.00)	3.00 (2.25–3.75)	0.428
Time from symptom onset to diagnosis (days)	3.50 (2.00–5.00)	2.00 (2.00–3.00)	0.414
Viral load (Ct value)	29.31 ± 4.33	29.67 ± 4.62	0.842
WBC ( × 10^9^/l; range: 3.5–9.5)	5.04 ± 1.44	5.03 ± 1.49	0.981
Lym ( × 10^9^/l; range: 1.1–3.2)	1.48 ± 0.56	1.80 ± 0.83	0.213
CD4 + T cell (/μl; range: 410–884)	476.00 (424.00–547.00)	645.00 (527.00–824.00)	0.126
CD8 + T cell (/μl; range: 190–658)	371.00 (326.00–437.00)	406.00 (345.25–470.00)	0.503
NK (/μl; range: 90–536)	324.00 (181.00–331.00)	277.00 (212.50–408.50)	0.37
Top body temperature (^o^C)	38.03 ± 0.75	38.01 ± 0.88	0.944
Pneumonia at admission	15 (88.24%)	9 (64.29%)	0.112
Severe pneumonia	2 (11.76%)	0 (0.00%)	0.185
Clinical type (mild/common/severe)	2/13/2	5/9/0	0.153
Duration of hospitalisation (days)	13.00 (9.00–18.00)	15.50 (13.00–16.75)	0.403

WBC, white blood cells; Lym, lymphocytes; NK, natural killer cells; G0, index cases; G1, first generation; G2, second generation; G3, third generation.

### Epidemiological and clinical characteristics in different generations among the family clusters

Among the family clusters, three index cases (G0) transmitted the infection to 14 first-generation cases (G1), who transmitted the infection to 12 second-generation cases (G2) and subsequently two third-generation cases (G3) (Figs 1 and 2). Then, the epidemiological and clinical characteristics were compared between generations 0 and 1 (G0 + G1) and generations 2 and 3 (G2 + G3) cases.

G0 + G1 cases were older than G2 + G3 cases (44.00 (42.00–55.00) *vs.* 37.00 (23.00–42.25) years, *P* = 0.039). The incubation time, the time from illness onset to hospitalisation, the time from illness onset to diagnosis and the duration of hospitalisation were not significantly different between the two groups (all *P* > 0.05). The proportion of mild cases in G2 + G3 cases (5/14, 35.71%) appeared to be larger than that in G0 + G1 cases (2/17, 11.76%) although the difference was not statistically significant (*P* = 0.153).

The viral loads were not significantly different between G0 + G1 and G2 + G3 cases (29.31 ± 4.33) *vs.* (29.67 ± 4.62), *P* > 0.05. The level of lymphocyte counts tended to be lower in G0 + G1 cases ((1.48 ± 0.56) × 10^9^/l) than in G2 + G3 cases ((1.80 ± 0.83) × 10^9^/l), but the difference was not statistically significant (*P* = 0.213) (Table 2).

### Comparison of the epidemiological and clinical characteristics between generations of the family clusters and sporadic cases

The differences of epidemiological and clinical characteristics between sporadic cases and generations of family clusters were determined. It was shown that the lymphocyte counts of sporadic cases were significantly lower than those of G2 + G3 cases (*P* = 0.033) but without a significant difference with G0 + G1 cases (*P* > 0.05). There were no differences in the incubation time, the time from illness onset to hospitalisation, the time from illness onset to diagnosis and the duration of hospitalisation among the three groups (*P* > 0.05) (Tables 1 and 2).

## Discussion

In this study, the epidemiological and clinical characteristics of three family clusters were investigated using sporadic patients as controls. Such a specific population provides us an opportunity to analyse the relationship between transmission and the disease presentation in different settings where the sporadic patients were used as external controls to the family cluster patients. More patients in sporadic cases came back from Wuhan than in the family cluster. All three index cases were latent patients without any symptoms at the time when they came back to Ruian. This study revealed that sporadic cases had lower levels of albumin and lymphocyte counts than family cluster cases; otherwise, there were no significant differences in terms of other epidemiological characters and clinical features between the two groups. In addition, the lymphocyte counts in sporadic cases were lower than those in the cases of second and third generations family cluster cases although there was no difference in the lymphocyte counts among the different generations within family cluster.

Human coronavirus pneumonia is often associated with an elevated production of chemokines, which recruit massive inflammatory cell infiltration and release cytokines resulting in acute pulmonary injury [13]. The decrease of lymphocyte counts and elevation of cytokines/chemokines are the hallmark of coronavirus-associated pneumonia and are associated with the severity of the pneumonia. Recent studies on COVID-19 have demonstrated that the lymphocyte counts in the peripheral blood are remarkably decreased in patients who are admitted in the intensive care unit (ICU), compared with non-ICU patients [1, 14]. In addition, several studies on COVID-19 or MERS have shown that hypoalbuminemia is a frequent feature and associated with the severity of the pneumonia [15–17], probably owing to increased energy consumption or altered pulmonary vessel permeability. The finding in this study that the decrease of lymphocyte counts and hypoalbuminaemia in sporadic cases, compared with family cluster cases, indicates that there is an increased immune activation or dysfunction and thus more severe pulmonary inflammation in sporadic cases than in family cluster cases.

Within the family clusters, virus transmission through different generations and the clinical presentations were investigated. The age of second- and third-generation patients was younger than that of the index and first-generation patients. It has been reported that when hosts of different sexes or ages were encountered, the pathogen may change optimal exploitative strategy, leading to considerable variation of pathogen transmission and virulence [18]. The trade-off between transmission and virulence would change in coordination with host immunity that is associated with age. SARS-CoV-2 seems prone to affect older males with comorbidities [19]. In this study, two patients, A1 (G1) and B1 (G1), developed severe pneumonia in first generation, while no severe cases were observed in the second or third generation. More mild patients were found in the second or third generation, which are also noted to be younger in age, may be attributed to age. In addition to, it could be inferred that following the passage through several generations within the family cluster, the virulence of SARS-CoV-2 decreased gradually.

Epidemiological evidence from COVID-19 family clusters has suggested that most index cases are asymptomatic carriers, mild patients or even latent patients [7, 9, 10], which is consistent with our observation. However, the characteristics of index cases with COVID-19 are reportedly different from those in the MERS family clusters, who have moderate or severe symptoms and are never asymptomatic carriers [20–22]. The higher virulence and mortality in MERS may explain the different characteristics of the index cases. As the viral load of SARS-CoV-2 detected in the asymptomatic patients is similar to that in the symptomatic patients [23], the asymptomatic index cases are capable of causing cluster infection in the family setting. A recent study with the largest sample so far in China showed that the median incubation period for COVID-19 was 4 days (IQR, 2−7), with the longest incubation period up to 24 days, and only 43.8% patients presented with a fever at admission [24], highlighting the importance of monitoring and isolating potential infected family members who have had an exposure history in the family setting.

Previous studies have demonstrated that the outbreak of SARS-CoV and MERS-CoV infections have resulted in large clusters of patients, most of which are associated with the nosocomial transmission, called super-spreader events [25–27]. Since the virological and clinical characteristics are similar among SARS-CoV, MERS-CoV and SARS-CoV-2 [28], it is worth noting the super-spreader events in COVID-19.

As of 7 March, the daily number of newly diagnosed patients had decreased to less than 150 cases in China. Currently, there are no specific antiviral agents or vaccines available for SARS-CoV-2, which possesses a high infectivity, and thus advanced epidemiological surveillance and timely identification and isolation of suspected cases or individuals who had a close contact or exposure history remains a priority to prevent family cluster or super-spread events. The Chinese experience shows that intensive social interventions, including isolation are crucial in delaying and blocking the spread and subsequent outbreaks of the disease.

In conclusion, family clusters of COVID-19 can be caused by latent patients. The epidemiological and clinical symptoms are similar between the family cluster and sporadic patients, but the sporadic patients showed lower lymphocytes and hypoalbuminaemia. These findings indicate that close surveillance, timely identification and isolation of the suspected or latent cases is crucial in preventing family clusters or even super-spread events.

## Data Availability

All data generated or analysed during this study are included in this paper.
